# Translation and psychometric evaluation of Smartphone Addiction Scale—Short Version (SAS-SV) among Chinese college students

**DOI:** 10.1371/journal.pone.0278092

**Published:** 2022-11-29

**Authors:** Hao Zhao, Shameem Rafik-Galea, Mimi Fitriana, Tian-Jiao Song

**Affiliations:** 1 School of Education, Shandong Women’s University, Jinan, Shandong, China; 2 Faculty of Education, Languages & Psychology, SEGI University, Kuala Lumpur, Malaysia; 3 Faculty of Arts and Science, International University of Malaya-Wales, Kuala Lumpur, Malaysia; National Cheng Kung University College of Medicine, TAIWAN

## Abstract

**Background:**

Smartphone addiction is very prevalent among college students, especially Chinese college students, and it can cause many psychological problems for college students. However, there is no valid research instrument to evaluate Chinese college students’ smartphone addiction.

**Objective:**

This study aimed to translate the Smartphone Addiction Scale—Short Version (SAS-SV) into Chinese and evaluate the psychometric characteristics of the Smartphone Addiction Scale- Chinese Short version (SAS-CSV) among Chinese college students.

**Methods:**

The SAS-SV was translated into Chinese using the forward-backward method. The SAS-CSV was completed by 557 Chinese college students (sample 1: n = 279; sample 2: n = 278). 62 college students were randomly selected from the 557 Chinese college students to be meas- ured twice, with an interval of two weeks. The reliability of the SAS-CSV was evaluated by internal consistency reliability and test-retest reliability, and the validity of the SAS-CSV was evaluated by content validity, structural validity, convergent validity, and discriminant validity.

**Results:**

The SAS-CSV presented good content validity, high internal consistency (sample 1: α = 0.829; sample 2: α = 0.881), and good test-retest reliability (ICC: 0.975; 95% CI: 0.966–0.985). After one exploratory factor analysis, three components (tolerance, withdrawal, and negative effect) with eigenvalues greater than 1 were obtained, and the cumulative variance contribution was 50.995%. The results of confirmatory factor analysis indicated that all the fit indexes reached the standard of good model fit (χ^2^/df = 1.883, RMSEA = 0.056, NFI = 0.954, RFI = 0.935, IFI = 0.978, TLI = 0.969, CFI = 0.978). The SAS-CSV presented good convergent validity for the factor loading of all the items ranged from 0.626 to 0.892 (higher than 0.50), the three latent variables’ AVE ranged from 0.524 to 0.637 (higher than 0.50), and the three latent variables’ CR ranged from 0.813 to 0.838 (higher than 0.70). Moreover, the square roots of the AVE of component 1 (tolerance), component 2 (withdrawal) and component 3 (negative effect) were 0.724, 0.778, and 0.798, respectively, higher than they were with other correlation coefficients, indicating that the SAS-CSV had good discrimination validity.

**Conclusion:**

The SAS-CSV is a valid instrument for measuring smartphone addiction among Chinese college students.

## Introduction

As the carrier in the development of mobile Internet, smartphones have become daily neces- sities for more and more people in modern society. Appropriate use of smartphones can sati- sfy people’s needs for entertainment, study, social contact, and shopping. However, the use of smartphones is a double-edged sword. Excessive use of smartphones can easily lead to smart- phone addiction [1]. According to some recent studies, smartphone addiction was defined as a condition where the use of smartphone has fulfilled a deep need (dependency, habitual, and addictive behavior) to the extent that the individual has difficulty conducting basic activities of daily life without the concurrent use of a smartphone, and as such caused neglect of other aspects of one’s life [2]. Previous studies have found smartphone addiction makes it difficult for college students to concentrate in class [3] and reduces their happiness in life [4–6]. In severe cases, smartphone addiction can lead to social anxiety [7], personality disorders [8], and suicidal tendencies [9–11] among college students. Due to the serious negative consequ- ences of smartphone addiction, it is thus important to concern smartphone addiction among college students.

To evaluate smartphone addiction, some scholars have developed some research instru- ments up to now, such as the Smartphone Addiction Scale (SAS) and Smartphone Addiction Scale—Short Version (SAS-SV) of Kwon et al. [12,13], the Smartphone Addiction Proneness Scale (SAPS) of Kim et al. [14], the Smartphone Addiction Inventory (SPAI) of Lin et al. [15], the Smartphone Addiction Scale for College Students (SAS-C) of Su et al. [16], the Smart- phone Addiction Scale for Chinese Adults (SAS-CA) of Chen et al. [17], and the Smartphone Application—Based Addiction Scale (SABAS) of Csibi et al. [18]. Among these research instruments for measuring smartphone addiction, the SAS-SV of Kwon et al. and the SABAS of Csibi et al. are widely used to measure smartphone addiction of college students. The SAS- SV has been verified in Turkey [19–22], Pakistan [23], Egypt [24], Britain [25], America [26], and China [27,28] as a good instrument to assess college students’ smartphone addiction. Moreover, in recent years, some scholars have paid more attention to the SABAS and applied it to evaluate the smartphone addiction of college students. The SABAS has been tested in Malaysia [29,30], Bangladesh [31], Indonesia [32], and China [30,33–36] as a good instru- ment to measure college students’ smartphone addiction.

According to the 48th China Statistical Report on Internet Development released by the China Internet Network Information Center (CINIC), as of June 2021, the scale of China’s mobile Internet users has reached 1007 million, and the proportion of Chinese Internet users who surf the Internet using smartphones is as high as 99.6%, among which the student group is the largest, accounting for 21% of the overall Internet users [37]. Some recent studies have shown that the incidences of Chinese college students’ “smartphone addiction” are also very high, which has been estimated at 18.6%–49.8% [38–43]. Although these studies actually wanted to focus on Chinese college students’ smartphone addiction, they used the scales that measured non-smartphone addiction to measure Chinese college students’ smartphone addi- ction, such as the Mobile Phone Addiction Index Scale (MPAIS) [44] and the Mobile Phone Addiction Tendency Scale (MPATS) [45]. These scales for measuring non-smartphone addi- ction ignore that smartphones are not only used for making phone calls and sending messages, and they do not fully consider the similar characteristics of smartphones and computers. Up to now, although the Smartphone Dependence Scale for College Students compiled by Su et al. can be directly used to measure college students’ smartphone addiction, its reliability and validity are not good enough [16]. The SAS-SV and SABAS have been verified by a few studies as good instruments to measure the smartphone addiction of college students in the Chinese mainland, but these studies only test the reliability of the two scales, and there is a lack of cross-cultural research on them [27,28,30]. Thus, there is currently a lack of a valid Chinese version of the smartphone addiction scale that can be directly used to measure the smartphone addiction of Chinese college students. In summary, it is very important to translate the SAS- SV or SABAS into Chinese and evaluate the psychometric characteristics of the Chinese versions of these two scales for exploring Chinese college students’ smartphone addiction.

The purpose of this study is to translate the SAS-SV into Chinese and evaluate the psycho- metric characteristics of the Smartphone Addiction Scale—Chinese Short Version (SAS-CSV) among Chinese college students, so as to provide a valid research instrument for further study of Chinese college students’ smartphone addiction.

## Methods and materials

### Design

This study was conducted at Shandong Women’s University and Shandong Normal University from May to June 2022, utilizing a quantitative approach. Firstly, the original SAS-SV was translated into Chinese. Then, the reliability of the SAS-CSV was evaluated by internal consi- stency reliability and test-retest reliability, and content validity, structural validity, convergent validity, and discriminant validity were used to evaluate the validity of the SAS-CSV.

### Linguistic validation

The SAS-SV was translated into Chinese using the forward-backward method [46,47]. The translation process included several steps: forward translation, discussion with translators, backward translation, expert discussion, and a cognitive test on purposefully selected sub- jects.

The SAS-SV was first translated into Chinese by Chinese native-speaker A, who spe- cializes in English applied linguistics and is a qualified translator, and then A discussed with the researcher to create an initial Chinese version. The initial Chinese version was then back translated into English by Chinese native-speaker B, who specializes in English applied linguistics and is a qualified translator. Later, the researcher invited two experts on smart- phone addiction to discuss and correct the ill-matched translations with A, B, and the rese- archer, thus forming the Smartphone Addition Scale—Chinese Short Version (SAS-CSV) (S1 File).

Given the COVID-19 pandemic, the researcher selected a class of 41 college students from Shandong Women’s University as participants by convenient sampling method to conduct item review on the SAS-CSV online. After the participants completed the online question- naire, the researcher interviewed the participants online from May 3, 2022, to May 7, 2022, for the following questions: (a) How much time did it take you to finish the questionnaire?

(b) Are the questionnaire instructions clear to you? If not, what is not clear? (c) Are there any questions unclear? If so, which one is unclear and what is unclear? (d) Are there any im- portant contents not covered in the questionnaire? (e) Do you have any suggestions for improving the questionnaire? According to the item review, the time for 41 participants to complete the questionnaire was 1–3 minutes, indicating that the number of questions in the questionnaire was appropriate. All the 41 participants reflected that the instructions and questions in the questionnaire were very clear and easy to understand.

### Sample and sampling procedure

In this study, 16 classes of college students from Shandong Women’s University and Shandong Normal University were selected as participants by cluster random sampling. With the help of class counselors, the researcher sought the consent of college students from these 16 classes to participate in the questionnaire survey through the QQ group. Given the COVID-19 pandemic, an online questionnaire containing an information sheet for participants and an informed consent form was distributed to the QQ group of the class by the QuizStar platform, and the students were informed that the online questionnaire was valid for two weeks (May 13, 2022 to May 26, 2022). Finally, 557 online questionnaires were collected, and all of the valid ones. The age range of all participants was 17–24 (M = 20.49; SD = 1.398). In this study, the whole sample was randomly divided into sample 1 (n = 279) and sample 2 (n = 278) (S1 Data). There was no significant difference between the smartphone addiction scores of sample 1 and those of sample 2 in demographic variables (Table 1), which indicated that the two samples were homogeneous in smartphone addiction scores. To test the reliability and validity of the SAS-CSV, sample 1 was used for internal consistency reliability analysis and exploratory factor analysis, and sample 2 was used for internal consistency reliability analysis, confirmatory factor analysis, convergent validity analysis, and discriminant validity analysis. It should be noted that in order to test the test-retest reliability of the SAS-CSV, 62 college students were randomly selected from the 557 Chinese college students to be measured twice at an interval of two weeks (S1 Data). Table 1 shows the sample characteristics of participants and comparison results of smartphone addiction scores of sample 1 and sample 2 in demographic variables.

**Table 1:** Sample characteristics of participants (n = 557) and comparison of smartphone addiction scores of sample 1 and sample 2 in demographic variables.

Variable	Categories	n (%)	Sample 1 (n = 279)		Sample 2 (n = 278)		p value
			n (%)	Mean (SD)	n (%)	Mean (SD)	
Gender	Male	245 (44%)	116 (41.6%)	37.310 (8.506)	129 (46.4%)	36.767 (11.061)	0.665
	Female	312 (56%)	163 (58.4%)	37.123 (8.038)	149 (53.6%)	37.195 (8.591)	0.939
Grade	Freshman	158	83 (29.7%)	36.904 (9.380)	75 (27.0%)	36.107 (9.771)	0.602
	Sophomore	135	78 (28.0%)	37.051 (6.289)	57 (20.5%)	37.070 (10.203)	0.990
	Junior	157	81 (29.0%)	38.321 (8.269)	76 (27.3%)	36.434 (10.968)	0.228
	Senior	107	37 (13.3%)	35.730 (8.928)	70 (25.2%)	38.500 (8.019)	0.105

### Statistical analysis

#### Content validity

This study used the content validity index (CVI) to evaluate the content validity of the SAS- CSV. Four experts were invited by the researcher to form a panel of experts to assess the content validity of the SAS-CSV and make recommendations for revisions, including two experts in developmental and educational psychology, one expert in psychometrics, and one expert in psychostatistics. The content validity of an item is acceptable when the CVI value is greater than or equal to 0.75 [48].

*Floor and ceiling effects*. The percentage of participants scoring in the upper decile of the scale was used to calculate the ceiling effect, while the percentage of participants scoring in the lower decile of the scale was used to calculate the floor effect. If more than 15% of parti- cipants have minimal or maximal scores, floor and ceiling effects are thought to be present [49]. Up to 25% were deemed moderate floor and ceiling effects, and more than 25% were deemed substantial [50].

*Internal consistency reliability*. The internal consistency reliability was evaluated by the Cronbach’s-α coefficient. When Cronbach’s-α coefficient is greater than 0.70, the reliability of the scale is acceptable [51].

*Test-retest reliability*. A retest was conducted two weeks after the baseline test. Evaluation by the Intraclass Correlation Coefficient (ICC) recommends 0.70 as the lowest reliability standard [52].

*Structural validity*. Exploratory factor analysis (EFA) and confirmatory factor analysis (CFA) were used to evaluate the instrument’s structural validity. With SPSS software (Version 19.0), the Kaiser-Meyer-Olkin (KMO) test, the Barlett Sphericity test, and EFA were perfor- med. The method of factor extraction was set as “Maximum Likelihood”, and the method of factor rotation was set as “Direct Oblimin”. CFA was performed with Amos software (Version 24.0). The χ^2^, degree of freedom (df), χ^2^/df, normed-fit index (NFI), relative fit index (RFI), incremental fit index (IFI), tucker-lewis index (TLI), comparative fit index (CFI) and root- mean-square error of approximation (RMSEA) were calculated to determine the fit indices. When the KMO value is greater than 0.5 and the Bartlett sphere test reaches a significant level (p < 0.05), the data can be subjected to EFA [51]. The criteria for good model fit are χ^2^/df < 3, CFI, TLI, and NFI values > 0.95, RFI, IFI values > 0.90, and RMSEA < 0.06 [53,54].

*Convergent and discriminant validity*. The convergent validity of the factor model was evaluated by factor loading of all items and all the latent variables’ average variance extracted (AVE). The criteria for good convergent validity are factor loading > 0.50 and AVE > 0.50 [51]. The discriminant validity was evaluated using the Fornell-Larcker criterion, which com- pares the square root of the AVEs with the correlation values between the factors. If the squ- are roots of the AVEs are larger than the correlations between the factors, then the model has good discriminant validity.

### Ethical consideration

This study was approved by the Research Ethics Committee of Shandong Women’s University. A written informed consent form was used in this study. The Research Ethics Committee of Shandong Women’s University approved the use of online informed consent form with letter reference number S/REC/04/56/2022 on April 19th, 2022. The online information sheet for participants introduced the research title, research purposes, risks and benefits, payment and compensation, confidentiality, etc. After the participants read the online information sheet, the online informed consent form would appear by clicking “Next”. If the participants agreed to participate in the study, they could sign and click “Next”, and the questionnaire would appear. All the participants in this study signed the online informed consent form.

## Results

### Content validity

Calculations revealed that the CVI value of other items was 1, except for item 8, which was 0.25, lower than 0.75. From the suggestions of the panel of experts, the low content validity item 8 “Constantly checking my smartphone so as not to miss conversations between other people on Twitter or Facebook” was revised to “constantly checking my smartphone so as not to miss conversations between other people on WeChat or QQ”. Then the panel of experts reevaluated the updated item’s content validity, and the outcome revealed that the CVI of item 8 was 1, thus the CVI value of all items of the SAS-CSV was 1.

### Psychometric properties of SAS-CSV

#### Floor and ceiling effects

In the sample 1 and sample 2, no floor effect or ceiling effect was found in the total score of SA and each dimension score. Specifically, the total score’s floor effect of sample 1 was 0.7%, and its ceiling effect was 0.4%. The total score’s floor effect of sample 2 was 0.7%, and its ceiling effect was 2.2%. For the sample 1, each dimension score’s floor effect ranged from 2.9% to 5.4%, and their ceiling effects ranged from 1.1% to 3.2%. For the sample 2, each dimension score’s floor effect ranged from 4.3% to 5.8%, and their ceiling effects ranged from 2.2% to 6.5% (Table 2). The above results demonstrated the good discrimination of the SAS-CSV.

**Table 2:** Cronbach’s-α coefficient, floor/ceiling effect, and mean and standard deviation of the dimensions of the Smartphone Addiction Scale—Chinese Short Version (SAS-CSV) among sample 1 (n = 279) and sample 2 (n = 278).

Dimensions of SAS-CSV	Cronbach’α		Floor/ceiling effect (%)		Mean (SD)	
	Sample 1	Sample 2	Sample 1	Sample 2	Sample 1	Sample 2
**Tolerance**	0.749	0.809	3.2/1.1	5.8/2.2	15.097 (3.798)	15.417 (4.072)
**Withdrawal**	0.762	0.827	5.4/3.2	4.3/6.5	11.043 (3.359)	10.824 (3.744)
**Negative effect**	0.718	0.809	2.9/3.2	4.7/6.5	11.061 (3.189)	10.755 (3.925)
**Total SAS-CSV**	0.829	0.881	0.7/0.4	0.7/2.2	37.201 (8.221)	36.996 (9.799)

**Key:-** SD = Standard Deviation.

#### Internal consistency reliability

For sample 1 and sample 2, the Cronbach’s-α coefficients of both the total scale and the subscales were greater than 0.70, indicating that the SAS-CSV had good internal consistency reliability. Specifically, for the sample 1, the Cronbach’s-α coe- fficient of the SAS-CSV was 0.829, and the Cronbach’s-α coefficients of the subscales ranged from 0.718 to 0.762. With regard to the sample 2, the Cronbach’s-α coefficient of the SAS- CSV was 0.881, and the Cronbach’s-α coefficients of the subscales ranged from 0.809 to 0.827 (Table 2).

#### Test-retest reliability

A sample of 62 college students was used to evaluate the test-retest reliability of the SAS-CSV. The result showed that the ICC of the SAS-CSV was 0.975 (95% CI: 0.966–0.985; p < 0.001), suggesting adequate test-retest reliability.

#### Structural validity

The KMO value was 0.826, greater than 0.8, and the Bartlett sphere test also reached the significance level (χ^2^ = 893.626; p < 0.001), indicating that this data was very appropriate for factor analysis.

Three components with eigenvalues greater than 1 were obtained after one EFA, and the cumulative variance contribution was 50.995%. Specifically, the eigenvalue of the first component was 3.989, and the first component accounted for 35.045% of the total variation. According to the common content explained by the four items (item 9, item 8, item 7, and item10), the first component was named “tolerance”. The eigenvalue of the second com- ponent was 1.449, and the second component accounts for 9.621% of the total variation. According to the common content explained by the three items (item 5, item 4, and item 6), the second component was named “withdrawal”. The eigenvalue of the third component was 1.019, and the third component accounts for 6.329% of the total variation. According to the common content explained by the three items (item 1, item 2, and item 3), the third com- ponent was named “negative effect” (Table 3).

**Table 3:** Exploratory factor analysis of the Smartphone Addiction Scale—Chinese Short Version (SAS-CSV) (n = 279).

SAS-CSV questions	Communality	Factor loading		
		Tolerance	Withdrawal	Negative effect
9. Using my smartphone longer than I had intended. 我使用智能手机的时间会超出预期	0.754	0.865		
7. I will never give up using my smartphone even when my daily life is already greatly affected by it. 即使我的日常生活已经受到智能手机的极大影响, 我也绝不会放弃使用智能手机	0.547	0.553		
8. Constantly checking my smartphone so as not to miss conversations between other people on WeChat or QQ. 我会不断检查我的智能手机, 以免错过其他人在微信或QQ上的对话	0.376	0.504		
10. The people around me tell me that I use my smartphone too much. 周围的人告诉我, 我使用智能手机太过度了	0.329	0.391		
Feeling impatient and fretful when I am not holding my smartphone. 当我没拿智能手机时, 我感到不耐烦和烦躁	0.556		0.733	
6. Having my smartphone in my mind even when I am not using it. 即使我不使用智能手机, 心里也会想着它	0.676		0.704	
4. Won’t be able to stand not having a smartphone. 无法忍受没有智能手机	0.385		0.583	
2. Having a hard time concentrating in class, while doing assignments, or while working due to smartphone use. 由于使用智能手机, 上课、做作业或工作时很难集中注意力	0.641			0.758
1. Missing planned work due to smartphone use. 由于使用智能手机, 错过了计划的工作	0.525			0.738
3. Feeling pain in the wrists or at the back of the neck while using a smartphone. 使用智能手机时感到手腕或脖子后面疼痛	0.311			0.461
Eigenvalue		3.989	1.449	1.019
Variance contribution (%)		35.045	9.621	6.329
Cumulative variance contribution (%)		35.045	44.666	50.995

Notes:- Extraction Method: Maximum Likelihood.

Rotation Method: Oblimin with Kaiser Normalization.

After CFA, the results showed that χ^2^/df, RMSEA, NFI, RFI, IFI, TLI, and CFI all reached the criteria (χ^2^/df = 1.883, RMSEA = 0.056 (90% CI: 0.034–0.078), NFI = 0.954, RFI = 0.935, IFI = 0.978, TLI = 0.969, CFI = 0.978), indicating that the SAS-CSV had good structural validity (Table 4).

**Table 4:** Fit indices of the Smartphone Addiction Scale—Chinese Short Version (SAS-CSV)’s structural equation model (n = 278).

Fitness index	χ^2^	df	p	χ^2^/df	RMSEA	RFI	IFI	CFI	TLI	NFI
Criteria			<0.05	<3	<0.06	>0.90	>0.90	>0.95	>0.95	>0.95
Model	60.243	32	0.002	1.883	0.056	0.935	0.978	0.978	0.969	0.954

**Key:-** df = Degree of Freedom, RMSEA = Root-Mean-Square Error of Approximation, RFI = Relative Fit Index, IFI = Incremental Fit Index, CFI = Comparative Fit Index, TLI = Tucker-Lewis Index, NFI = Normed-Fit Index.

### Convergent and discriminant validity

**Fig 1:**
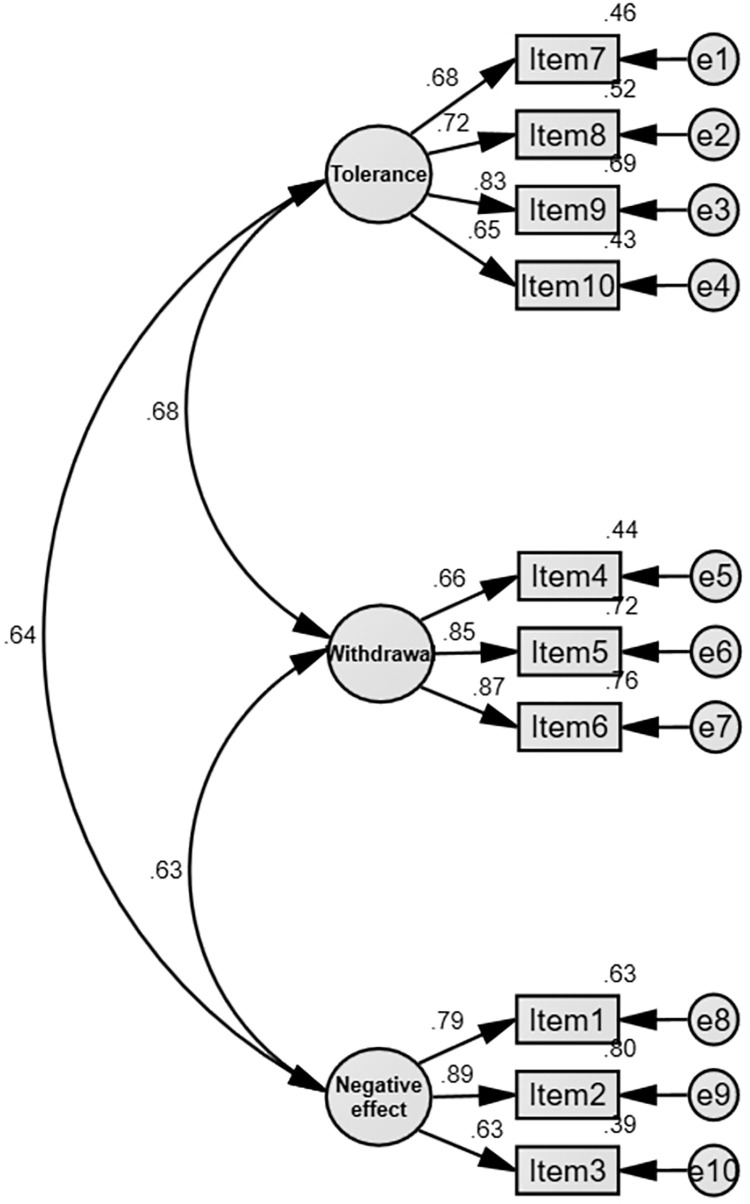
Smartphone Addiction Scale—Chinese Short Version (SAS-CSV)’s three-dimension model.

As for the convergence validity, the factor loading of all the items ranged from 0.626 to 0.892, which is higher than 0.50. The three latent variables’ AVE ranged from 0.524 to 0.637, higher than 0.50. The three latent variables’ CR ranged from 0.813 to 0.838, higher than 0.70. The above results demonstrated that the scale had good convergence validity (Table 5 and Fig 1).

**Table 5:** Convergence validity of the Smartphone Addiction Scale—Chinese Short Version (SAS-CSV).

Factors	Item	Unstd.	S.E.	Z	P	Std.	C.R.	AVE
**Tolerance**	7. I will never give up using my smartphone even when my daily life is already greatly affected by it. 即使我的日常生活已经受到智能手机的极大影响, 我也绝不会放弃使用智能手机	1				0.677	0.813	0.524
	8. Constantly checking my smartphone so as not to miss conversations between other people on WeChat or QQ. 我会不断检查我的智能手机, 以免错过其他人在微信或QQ上的对话	1.048	0.102	10.251	***	0.721		
	9. Using my smartphone longer than I had intended. 我使用智能手机的时间会超出预期	1.148	0.104	11.044	***	0.829		
	10. The people around me tell me that I use my smartphone too much. 周围的人告诉我, 我使用智能手机太过度了	0.966	0.102	9.459	***	0.655		
**Withdrawal**	Missing planned work due to smartphone use. 由于使用智能手机, 错过了计划的工作	1				0.794	0.819	0.606
	Having a hard time concentrating in class, while doing assignments, or while working due to smartphone use. 由于使用智能手机, 上课、做作业或工作时很难集中注意力	1.107	0.078	14.155	***	0.892		
	Feeling pain in the wrists or at the back of the neck while using a smartphone. 使用智能手机时感到手腕或脖子后面疼痛	0.759	0.073	10.35	***	0.626		
**Negative effect**	Won’t be able to stand not having a smartphone. 无法忍受没有智能手机	1				0.661	0.838	0.637
	Feeling impatient and fretful when I am not holding my smartphone. 当我没拿智能手机时, 我感到不耐烦和烦躁	1.168	0.101	11.539	***	0.846		
	Having my smartphone in my mind even when I am not using it. 即使我不使用智能手机, 心里也会想着它	1.208	0.104	11.601	***	0.870		

**Key:-** Std. = Standard, Unstd. = Unstandard, S.E. = Standard Error, C.R. = Composite Reliability, AVE = Average Variance Extracted.

*** *p* < 0.001.

With regard to discriminant validity, this study found that the square root of the AVE of component 1 was 0.724, larger than the correlation coefficient between component 1 and the other two components. The square root of the AVE of component 2 was 0.778, larger than the correlation coefficient between component 2 and the other two components. The square root of the AVE of component 3 was 0.798, larger than the correlation coefficient between com- ponent 3 and the other two components (Table 6).

**Table 6:** Discriminant validity of the Smartphone Addiction Scale—Chinese Short version (SAS-CSV).

Factors	Tolerance	Withdrawal	Negative effect
**Tolerance**	**0.724**		
**Withdrawal**	0.645	**0.778**	
**Negative effect**	0.630	0.677	**0.798**

**Bold values:** Square root of average variance extracted.

## Discussion

The purpose of this study was to translate the SAS-SV and make it a valid instrument to evaluate Chinese college students’ smartphone addiction. The results indicated that the SAS-CSV had good psychometric properties in reliability and validity.

In this study, the forward-backward method was used to translate the SAS-SV into Chinese, and then the panel of experts evaluated the content validity of 10 items in the SAS-CSV. Acc- ording to the suggestions of the panel of experts, the researcher revised item 8, and then the panel of experts re-evaluated the content validity of item 8. The final CVI value of all items of the SAS-CSV was 1, indicating that the SAS-CSV had good content validity. The SAS-CSV showed good content validity, which may be explained by the fact that in the original study, 10 of the 33 questions from the SAS were identified by 7 experts based on content validity evaluation as the ones that best assessed smartphone addiction [13].

With regard to the reliability of the SAS-CSV, the results showed that the SAS-CSV had good internal consistency reliability and test-retest reliability. Cronbach’s-α coefficients for both the total scale and the subscales were greater than 0.70 in either sample 1 or sample 2. The Cronbach’s-α coefficients (sample1: 0.829; sample 2: 0.881) of the total scale were close to the study conducted in Iran (Cronbach’s-α of 0.85) for the Iranian version of SAS-SV [55], Serbia (Cronbach’s-α of 0.89) for the Serbian Version of SAS-SV [56], Spain (Cronbach’s-α of 0.88) for the Spanish version of SAS-SV [57], France (Cronbach’s-α of 0.90) for the French version of SAS-SV [57], and Turkey (Cronbach’s-α of 0.867) for the Turkish version of SAS-SV [19], all of which were higher than 0.80. The test-retest reliability test in this study found that the SAS-CSV had good test-retest reliability (ICC: 0.975; 95% CI: 0.966–0.985; p < 0.001), and the ICC of the SAS-CSV was almost in line with the study conducted in Serbia’s study (ICC: 0.94; 95% CI: 0.92–0.96) [56] and Turkey’s study (ICC: 0.926) [19]. The reason for the good reliability of the SAS-CSV may be that the researcher adhered rigorously to the translation process to guarantee translation correctness.

Regarding the structural validity of the SAS-SV, EFA and CFA were not carried out in the original study [13] and CFA was not carried out in the cross-cultural studies of the SAS-SV [19,56–58]. Specifically, in the original study, Kwon et al. only evaluated the internal consistency reliability and concurrent validity of the SAS-SV but did not use EFA and CFA to test the structural validity of the SAS-SV [13]. As for the cross-cultural studies on the SAS- SV, many studies only examined the internal consistency reliability of the scale and the structural validity of the scale through EFA, but these studies did not use CFA to examine the structural validity of the scale [19,56–58]. Given the common deficiencies of these studies, the researcher conducted EFA and CFA in this study. The results of EFA revealed that the SAS-CSV contains three factors, which together explained the variance variation of 50.995%. As opposed to the SAS-SV with unidimensional factor structure, the SAS-CSV with three- factor structure is more suitable for measuring smartphone addiction among Chinese college students. The eigenvalue of the first factor “tolerance” was 3.989, which contains four items, accounting for 35.045% of the total variance variation. Tolerance was defined as always trying to control one’s smartphone use but always failing to do so [12]. The eigenvalue of the second factor “withdrawal” was 1.449, which contains three items, accounting for 9.621% of the total variance variation. Withdrawal refers to impatience, restlessness and intolerability in the absence of a smartphone, the constant presence of one’s smartphone in one’s mind even when not using it, never giving up using one’s smartphone, and becoming irritable when being disturbed while using it [12]. The eigenvalue of the third factor "negative effect" was 1.019, which contains three items, accounting for 6.329% of the total variance variation. In this study, negative effect was defined as the impairment of physical, psychological, and social func- tioning of individuals due to excessive use of smartphones. Compared with the Smartphone Addiction Scale (SAS) of Kwon et al. [12], the Smartphone Addiction Proneness Scale (SAPS) of Kim et al. [14], and the Smartphone Addiction Inventory (SPAI) of Lin et al. [15], we found that the SAS-CSV had the same factors (tolerance and withdrawal) as these scales. Moreover, the negative effect measures not only the functional impairment of smartphone addiction that the SAS, SAPS, and SPAI are designed to evaluate, but also the physical harm caused to individuals by smartphone addiction. The results of CFA showed that the fit indexes of the SAS-CSV’s SEM all reached the standard of good model fit. For example, CFI, TLI, and NFI were all greater than 0.95, RFI and IFI were both greater than 0.90, and RMSEA was less than 0.06. The good structural validity of the SAS-CSV may be due to the fact that this study used an appropriate sampling method to extract a representative sample and split it randomly into two samples for the EFA and CFA, and the smartphone addiction scores of these two samples were found to be homogeneous in demographic variables by testing.

Convergent validity analysis and discriminant validity analysis were performed to conf- irm the good validity of the SAS-CSV from multiple perspectives. Firstly, the following findings verified that the SAS-CSV had good convergent validity: (a) The factor loadings of all the items ranged from 0.626 to 0.892, which were higher than 0.50. (b) The three latent variables’ AVE ranged from 0.524 to 0.637, higher than 0.50. (c) The three latent variables’ CR ranged from 0.813 to 0.838, higher than 0.70. Few previous cross-cultural studies on the SAS-SV have evaluated the convergent validity of the SAS-SV, and the few studies have evaluated the convergent validity of the scale from only one perspective. For example, Andrade et al. conducted Pearson correlation analysis of the SAS-SV total score and smart- phone use to evaluate the convergent validity of the Brazilian version of SAS-SV [59]. Nikolic et al. calculated the Pearson correlation coefficient of the SAS-SV total score and time indicators of smartphone use to evaluate the convergent validity of the Serbian version of SAS-SV [56]. Different from the studies of Andrade et al. and Nikolic et al., this study provides stronger evidence of good convergent validity of the SAS-CSV from three pers- pectives (factor loading, latent variables’ AVE, and latent variables’ CR). Then, regarding discriminatory validity, this study found the square root of the AVE of component 1 was 0.724, larger than the correlation coefficient between component 1 and the other two components; similarly, the square roots of the AVEs of component 2 and component 3 were 0.778 and 0.798, respectively, greater than they were with other correlation coefficients. The result indicated the good discriminant validity of the SAS-CSV, suggesting that the three factors of the SAS-CSV are relatively independent, further demonstrating that the SAS-CSV is a multi- dimensional scale rather than a unidimensional scale. Therefore, we can evaluate Chinese college students’ smartphone addiction not only in the SAS-CSV total score but also in the three dimensions: tolerance, withdrawal, and negative effect, respectively.

## Conclusion and recommendation

The SAS-CSV was good for content validity, internal consistency reliability, test-retest reliability, structural validity, convergent validity, and discriminant validity, so the SAS-CSV is a valid instrument for measuring smartphone addiction of Chinese college students. On the basis of this research instrument, future research needs to further explore the causes of Chinese college students’ smartphone addiction and effective intervention methods. Also, it is very important to prevent and intervene in college students’ addiction to smartphones from a public health perspective. This instrument may be an important tool in the decision-making process, especially when implementing educational interventions and planning or implemen- ting public policies for smartphone-addicted college students.

## Supporting information

S1 File Smartphone Addiction Scale—Chinese Short Version. (DOCX)

S1 Data Data for evaluating the SAS-CSV. (XLSX)
