# Supplementary material for: Translation and psychometric evaluation of Smartphone Addiction Scale—Short Version (SAS-SV) among Chinese college students
Source: PLoS One. 2022 Nov 29;17(11):e0278092. doi: 10.1371/journal.pone.0278092 (PMC9707792; doi:10.1371/journal.pone.0278092)
Supplement: S1 File — (DOCX) [file pone.0278092.s001.docx]

**智能手机成瘾量表—中文简版**

为了调查大学生智能手机的使用状况，现需要您填写下面的调查问卷。以下问卷中的问题是您熟悉的内容，一共有6个选项: 1为非常不同意；2为不同意；3为有点不同意；4为有点同意；5为同意，6为非常同意，请勾选相应的选项。

|  | 非常不同意 | 不同意 | 有点不同意 | 有点  同意 | 同意 | 非常  同意 |
| --- | --- | --- | --- | --- | --- | --- |
|  | 1 | 2 | 3 | 4 | 5 | 6 |
| 1. 由于使用智能手机，错过了计划的工作。 |  |  |  |  |  |  |
| 1. 由于使用智能手机，上课、做作业或工作时很难集中注意力。 |  |  |  |  |  |  |
| 1. 使用智能手机时感到手腕或脖子后面疼痛。 |  |  |  |  |  |  |
| 1. 无法忍受没有智能手机。 |  |  |  |  |  |  |
| 1. 当我没拿智能手机时，我感到不耐烦和烦躁。 |  |  |  |  |  |  |
| 1. 即使我不使用智能手机，心里也会想着它。 |  |  |  |  |  |  |
| 1. 即使我的日常生活已经受到智能手机的极大影响，我也绝不会放弃使用智能手机。 |  |  |  |  |  |  |
| 1. 我会不断检查我的智能手机，以免错过其他人在微信或QQ上的对话。 |  |  |  |  |  |  |
| 1. 我使用智能手机的时间会超出预期。 |  |  |  |  |  |  |
| 1. 周围的人告诉我，我使用智能手机太过度了。 |  |  |  |  |  |  |
